# Control of Flowering and Cell Fate by LIF2, an RNA Binding Partner of the Polycomb Complex Component LHP1

**DOI:** 10.1371/journal.pone.0016592

**Published:** 2011-01-31

**Authors:** David Latrasse, Sophie Germann, Nicole Houba-Hérin, Emeline Dubois, Duyen Bui-Prodhomme, Delphine Hourcade, Trine Juul-Jensen, Clémentine Le Roux, Amel Majira, Nathalie Simoncello, Fabienne Granier, Ludivine Taconnat, Jean-Pierre Renou, Valérie Gaudin

**Affiliations:** 1 Institut J.-P. Bourgin, UMR1318 INRA-AgroParisTech, INRA Centre de Versailles-Grignon, Versailles, France; 2 Centre Léon Bérard, Inserm U590, Oncogenèse et progression tumorale, Lyon, France; 3 Centre de Génétique Moléculaire, CNRS FRE3144, Gif-sur-Yvette, France; 4 Biologie du Fruit, UMR 619 INRA Centre de Bordeaux, Villenave-d'Ornon, France; 5 Unité de Recherche en Génomique Végétale, Evry, France; University of Georgia, United States of America

## Abstract

Polycomb Repressive Complexes (PRC) modulate the epigenetic status of key cell fate and developmental regulators in eukaryotes. The chromo domain protein LIKE HETEROCHROMATIN PROTEIN1 (LHP1) is a subunit of a plant PRC1-like complex in *Arabidopsis thaliana* and recognizes histone H3 lysine 27 trimethylation, a silencing epigenetic mark deposited by the PRC2 complex. We have identified and studied an LHP1-Interacting Factor2 (LIF2). LIF2 protein has RNA recognition motifs and belongs to the large hnRNP protein family, which is involved in RNA processing. LIF2 interacts *in vivo*, in the cell nucleus, with the LHP1 chromo shadow domain. Expression of *LIF2* was detected predominantly in vascular and meristematic tissues. Loss-of-function of *LIF2* modifies flowering time, floral developmental homeostasis and gynoecium growth determination. *lif2* ovaries have indeterminate growth and produce ectopic inflorescences with severely affected flowers showing proliferation of ectopic stigmatic papillae and ovules in short-day conditions. To look at how LIF2 acts relative to LHP1, we conducted transcriptome analyses in *lif2* and *lhp1* and identified a common set of deregulated genes, which showed significant enrichment in stress-response genes. By comparing expression of LHP1 targets in *lif2*, *lhp1* and *lif2 lhp1* mutants we showed that *LIF2* can either antagonize or act with *LHP1*. Interestingly, repression of the *FLC* floral transcriptional regulator in *lif2* mutant is accompanied by an increase in H3K27 trimethylation at the locus, without any change in LHP1 binding, suggesting that LHP1 is targeted independently from LIF2 and that LHP1 binding does not strictly correlate with gene expression. LIF2, involved in cell identity and cell fate decision, may modulate the activity of LHP1 at specific loci, during specific developmental windows or in response to environmental cues that control cell fate determination. These results highlight a novel link between plant RNA processing and Polycomb regulation.

## Introduction

In eukaryotes, cell-fate determination, differentiation and developmental programs require precise spatial and temporal control of gene expression. Balance between gene activation and repression, as well as mechanisms of maintenance and erasure of expression patterns, require fine gene tuning. Polycomb group (PcG) proteins are key transcriptional regulators in these mechanisms [1]–[4]. PcG proteins are structurally diverse proteins assembled into chromatin-associated multi-protein complexes which cooperatively establish silent chromatin states [2], [5]. Studies in *Drosophila* described at least three main types of complexes with different functions which serve as reference types in other species: Polycomb repressive complex 1 (PRC1), PRC2, and the Pho repressive complex (PhoRC). The DNA binding factor (Pho) of PhoRC has a tethering function to initiate the recruitment of other PcG complexes. The PRC2 complex is involved in the trimethylation of the Histone H3 lysine 27 residue (H3K27me3), which is recognized by the chromo domain of Polycomb (Pc), one of the subunits of the PRC1 complex. Besides Pc, the core PRC1 is composed by three other conserved subunits, named dRing, Posterior sex combs (Psc) and Polyhomeotic (Ph) in *Drosophila* [6]. The PRC1 subunits containing a RING-finger domain (dRing, Psc and their related proteins) lead to the monoubiquitination of histone H2A (H2AK119ub), a histone modification associated with transcriptional repression.

Accumulating evidence indicates a larger diversity of PcG complexes than originally expected, providing functional flexibility. The diversity is mainly achieved by the incorporation of different homologues of core subunits, and through interactions with additional PcG proteins involved in recruiting the complexes or in modulating their repressive activities [7]. Furthermore, the genome-wide mapping of several PcG proteins on chromosomes revealed a large number of gene targets, in agreement with their increasing roles in various processes such as cell cycle regulation, stem cell self-renewal, genomic imprinting and developmental control. Because most of the PRC complexes (except PhoRC) lack specific DNA binding components, an important issue is how specificity in recruitment to loci is achieved and what components are involved. Some studies suggested that besides chromatin-associated factors, long non-coding RNA might also act as PcG recruiters [8]–[10]. Therefore, the diverse set of PcG complexes still awaits characterization.

In plants, the PRC2 components are the best-described PcG proteins due to their good conservation, both in terms of structure and function, to animals. Most of the PRC2 core subunits have several plant homologues suggesting the existence of a family of plant PRC2 complexes. Three PRC2 complexes have been already described and these act mainly in different developmental phases [11], [12]. As expected, mutations in PRC2 core components induce various perturbations of developmental programs (embryogenesis, flowering time, vernalization), cell proliferation, or cell identity [13]–[18].

The existence of a plant PRC1-like complex was for a long time a matter of debate, but recent studies demonstrate its existence. Firstly, evidence was provided by the recognition of H3K27me3 by LIKE HETEROCHROMATIN PROTEIN1 (LHP1), as Polycomb, its functional homologue in the PRC1 [19], [20]. Genome-wide distribution analysis revealed that LHP1 binds to several hundreds of sharply defined euchromatic genomic regions associated with H3K27me3 [20]. The LHP1 chromatin profiling is more similar to mammal PcG distribution with tight colocalization of PcG and H3K27me3, rather than to PcG distribution in *Drosophila* [21]. Mainly located in euchromatin [20], [22], LHP1 was shown to maintain chromatin repression at specific loci encoding transcription factors [23]–[25]. Mutations in *LHP1* cause changes in inflorescence development, alterations in leaf morphogenesis and cell size, and affect flowering time [26]–[29]. *lhp1* mutants are early flowering and have curled leaf morphology [26] thus sharing some phenotypic traits with *curly leaf* (*clf*) mutants affected in a PRC2 core component.

A second set of evidence was provided by studies on the putative PRC1 RING-finger homologs in *A. thaliana*. Four RING-finger proteins (AtRING1a, AtRING1b, AtBMI1A and AtBMI1B) were shown to interact *in vitro* with LHP1 and are involved in gene repression [30]–[32]. Mutations in *AtRING1a/b* cause ectopic meristem formation in cotyledons and leaves, floral homeotic conversions of anther-like and formation of stigma-like structures on floral organs [30], [32]. Mutations in *AtBMI1A/B* cause ectopic embryo formation and cell identity perturbations [31], [32].

The last evidence came from demonstration that H2A monoubiquitination occurs also in plants and that it is mediated by AtBMI1A/B activity [31]. Other proteins may also participate to a PRC1-like complex, such as EMF1 protein, which is involved in floral repression during the vegetative phase, interacts with AtBMI1A/B proteins, and may participate to H2A monoubiquitination regulation [31], [33]. To complete the conservation with animal PRC1, loss-of-function of plant PRC1 components reveal essential roles in regulating cell fate decision and proliferation [28], [30]–[32].

There is a large number of LHP1 targets. Furthermore, LHP1 colocalizes *in vivo* mainly with H3K27me3, yet the LHP1 chromo domain shows similar affinity for H3K9me2 and H3K27me3 *in vitro* [20]. This raises the hypothesis that different chromatin effectors interact with LHP1 to specify *in vivo* LHP1 targeting or its function. A C-terminal protein-protein interaction domain present in LHP1, the chromo shadow domain, may serve as a platform between LHP1 and numerous partners. Plants might thus have evolved aspects of gene regulation diversity by incorporating a multifunctional PRC1-like subunit, thus compensating for having a unique *LHP1* gene compared to 3 to 5 in animals.

Until now, only a few proteins have been identified which interact *in vitro* with LHP1 [30], [31], [34]–[40]. To further investigate LHP1 regulation, we searched for LHP1-Interacting Factors (LIF). We identified LIF2, a putative RNA-binding protein (RBP) of the large hnRNP family. We performed analyses of *LIF2* expression and its protein subcellular localization. Loss-of-function *lif2* mutants show a mild-early flowering phenotype compared to *lhp1* but have various floral developmental defects. In flowers grown in restrictive short-day conditions, the *lif2* fourth whorl produced ectopic inflorescences with aberrant flowers. These data suggest that *LIF2* participates in robust floral development in response to external conditions and in cell fate decision. Combined transcriptome analyses in *lif2* and *lhp1* mutants revealed that the class of deregulated genes in both mutants is enriched for stress-response genes. Expression and chromatin analyses revealed that LIF2 can antagonize or act similarly to LHP1 without displacing LHP1. Our data suggest that LIF2 and LHP1 act in overlapping pathways linking developmental control and environmental signaling pathways, with LIF2 possibly modulating LHP1 activity on a subset of LHP1 targets, in responses to these external cues.

## Results

### LIF2, an RRM containing protein, interacts with the chromo shadow domain of LHP1

**Figure 1:**
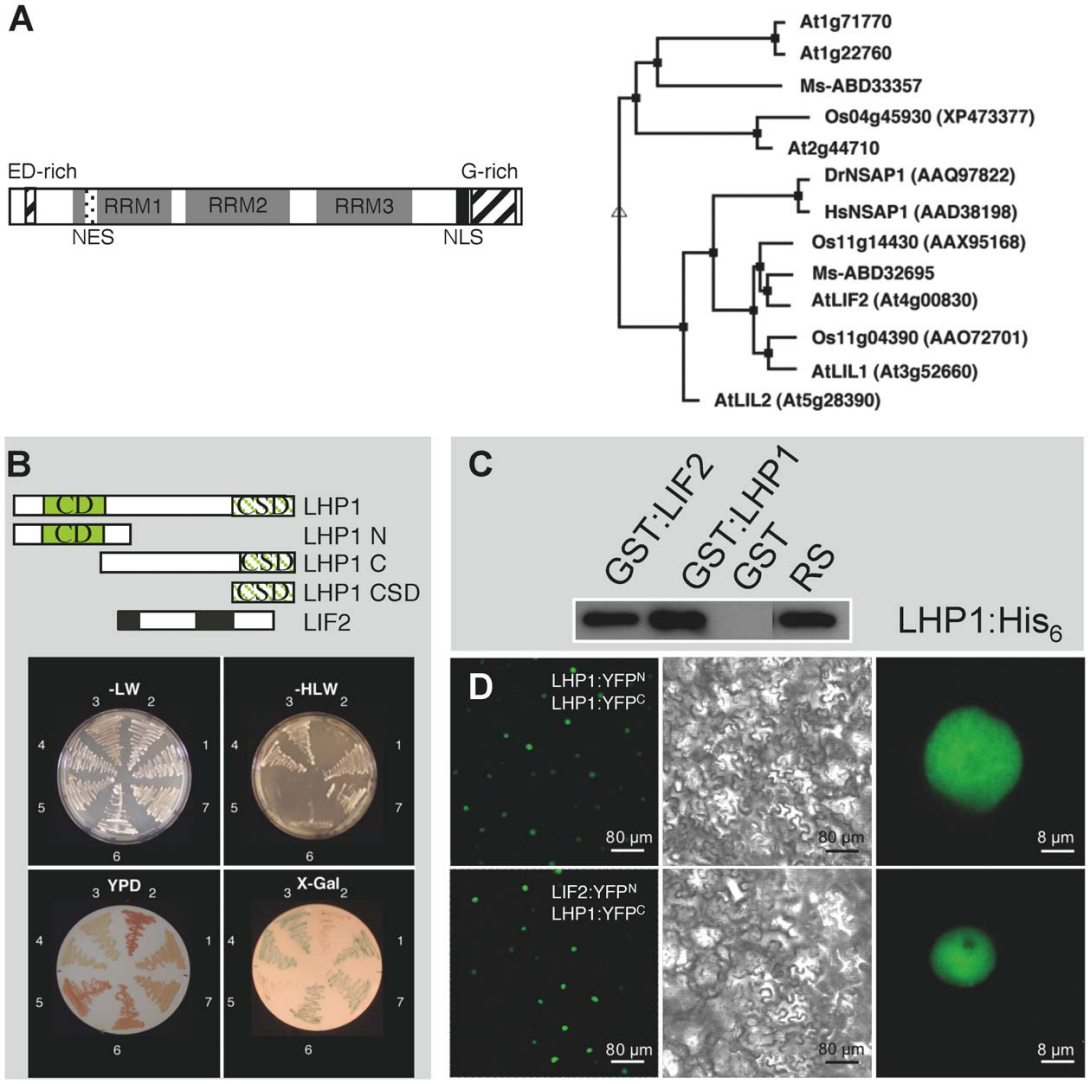
LHP1 interacts with the RRM containing protein LIF2 *in vitro* and *in vivo*. (A) LIF2 structure and related proteins. NLS (dotted box), NES (black box), RRMs 1 to 3 (gray boxes), glutamic acid and aspartic acid (ED) -rich domain and glycine (G) -rich domain (hatched boxes). The phylogenetic tree was generated with the MultAlign program (Risler-50-0 parameters) (http://bioinfo.genotoul.fr/multalin/). *At*: *A. thaliana*; *Ms: Medicago sativa*; Os: *Oryza sativa*; Hs: *Homo sapiens*. (B) LIF2 interactions with LHP1 in yeast two-hybrid assays. 1, pBD-LHP1 and pAD-LIF2. 2, pBD-LHP1N and pAD-LIF2. 3, pBD-LHP1C and pAD-LIF2. 4, pBD-LHP1CSD and pAD-LIF2. 5, pTD1-1, encoding p53 (negative control) and pAD-LIF2. 6, pBD and pAD. 7, pBD-LHP1 and pAD-LHP1 (positive controls). Selective media lacking leucine (L), tryptophan (W) or histidine (H) and rich YPD medium were used. On YPD medium, interacting and non-interacting proteins resulted in white and red colonies, respectively. β-galactosidase activity was performed on X-Gal plates. (C) Pull-down assay with the LHP1:His_6_ fusion and the GST:LIF2, GST:LHP1 or GST proteins. 2% of the amount of LHP1:His_6_ used in the binding assay was loaded as reference sample (RS). (D) BiFC experiments. Upper panel: LHP1 dimerization in the nucleus. Lower panel: Nuclear interactions between LHP1 and LIF2. Epifluorescence (left and right panels) and bright field (middle panel) CLSM images.

We searched for LHP1-INTERACTING FACTORs (LIF), partners of the LHP1 chromatin protein, by carrying out a yeast two-hybrid screen, using the full length LHP1 protein as bait and an *Arabidopsis* cDNA expression library. We identified 37 interacting proteins, most of them with unknown function. One of these proteins, LIF2 (At4g00830), contains three well-conserved RNA recognition motifs (RRM) [41], [42], a C-terminal nuclear localization signal (NLS), and two auxiliary domains with unusual amino acid distributions (glutamic acid and aspartic acid rich region; a glycine-rich region) (Fig. 1A). The C-terminal glycine-rich domain has a putative RGG box (arginine- and glycine-rich), a motif also present in RNA binding proteins [43]. The LIF2 structure strongly suggested an RNA-binding activity, opening interesting and unexplored links between RNA metabolism and LHP1-mediated regulation we wished to further investigate. Two *Arabidopsis* genes were identified as closely-related to *LIF2*: *LIL1* (*LIF2-like 1*, At3g52660) and *LIL2* (At5g28390). *LIL1* and *LIL2* encode proteins displaying 42.2% and 37.9% amino acid identity to LIF2, respectively. Closely-related LIF2 proteins are also present in *Medicago sativa* and *Oryza sativa*. Homologies with animal proteins suggest that LIF2 belongs to the large family of heterogeneous nuclear ribonucleoproteins (hnRNPs) implicated in diverse steps of RNA processing (Fig. 1A).

Interactions between LIF2 and LHP1 were investigated further by searching for interaction domains. In animals, many of the partners of HP1 contain the PxVxL consensus pentapeptide, which mediates interactions with the chromo shadow domain (CSD) of HP1 proteins [44]–[48]. Analysis of the sequence of LIF2 revealed the presence of a potential pentapeptide motif at the end of RRM3 (^361^LEVVL^365^). The mutagenesis or deletion of this putative pentapeptide did not abolish LIF2 and LHP1 interactions in yeast two-hybrid assays (data not shown). Using truncated LHP1 proteins [26], we showed that the chromo shadow domain (CSD) of LHP1 was necessary and sufficient for LIF2 interaction (Fig. 1B). The *LIF2* clone isolated in our screen corresponded to a partial cDNA (aa 204–495), showing that the LIF2 C-terminal region is sufficient for interaction with LHP1.

### LIF2 interacts with LHP1 *in vitro* and *in planta*

We tested the *in vitro* interaction between LIF2 and LHP1. By using LHP1 protein fused to a His_6_ tag and LIF2 protein fused to GST, we could confirm the interaction by *in vitro* pull-down assays (Fig. 1C). Interaction between His_6_:LHP1 and GST:LHP1 fusion proteins was also shown to occur (Fig. 1C) as expected from previous two hybrid assays [26], while no interaction was found with GST alone.

The LHP1-LIF2 *in vivo* interaction was analyzed by bimolecular fluorescence complementation (BiFC) experiments *in planta* [49]. The *LIF2* and *LHP1* cDNAs were inserted into the binary vectors, containing the split YFP N-terminal fragment (YFP^N^) and the C-terminal fragment (YFP^C^) and their expression was analyzed in transiently transformed *N. benthamiana* leaves [50]. We firstly confirmed that the LHP1 dimerization occurred *in viv*o in the plant nucleus (Fig. 1D). When the LIF2:YFP^N^ and LHP1:YFP^C^ proteins were expressed together, uniform YFP fluorescence was observed throughout the nucleoplasm. Similar results were obtained when expressing the LIF2:YFP^C^ and LHP1:YFP^N^ fusion proteins (data not shown). As expected, no fluorescence was detected when each tagged protein was expressed separately (data not shown). Thus, LIF2 and LHP1 interact *in vivo*, in the nucleus of plant cells.

### LIF2 expression patterns and protein localization

**Figure 2:**
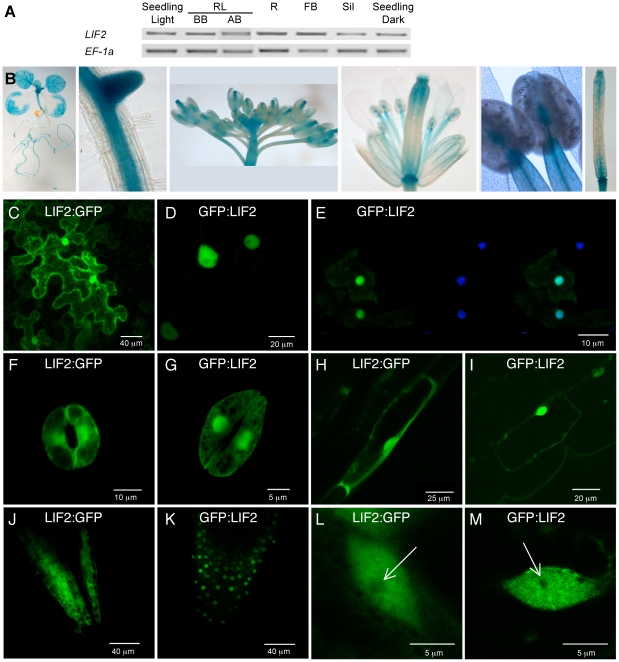
*LIF2* expression and localization of the LIF2 protein. (A) Semi-quantitative RT-PCR showing *LIF2* mRNA levels in ten-day-old *in vitro* seedlings grown under light or dark conditions, in rosette leaves (RL) before (BB) and after (AB) bolting, in roots (R), floral buds (FB) and siliques (Sil). The expression of *EF-1α* was used as a control. (B) Activity of *LIF2* promoter monitored by *GUS* expression patterns (blue color) in transgenic *proLIF2::GUS* plants. From left to right: 14-day-old seedling, root with a lateral primordium, inflorescence, single flower, anthers, gynoecium. (C–M) Subcellular localization of the GFP:LIF2 and LIF2:GFP fusion proteins. (C–D) Transient assay in *N. benthamiana* epidermal leaf cells. (E) Transient assay in *Arabidopsis* seedlings with from left to right, GFP fluorescence, DAPI counterstaining (for nuclei) and overlay. (F–M) Localization in stable *Arabidopsis* transgenic plants: (F–G) in guard cells, (H–I) hypocotyl cells, (J–K) root apex, and (L–M) root hair cells (the arrow indicates the nucleolus position).

To understand when and where *LIF2* acted, we studied its expression by RT-PCR analyses. *LIF2* was expressed throughout development in roots, leaves, floral buds and siliques (Fig. 2A). We then examined the *LIF2* expression pattern using transgenic lines expressing the *GUS* reporter gene under the control of its own promoter (i.e. 3 kb upstream of the Start codon) (Fig. 2B). In young plants, the pro*LIF2::GUS* construct was expressed in the distal regions of cotyledons, throughout leaves and root apical meristem, lateral root meristems and young floral buds. A strong expression was detected in the vascular tissues of various organs (root, leaf, hypocotyl, sepal, petal, anther filament) as well as in the gynophore and gynoecium (Fig. 2B). In the gynoecium, expression was mainly detected in apical and basal regions as well as in the developing ovules in these regions (Fig. 2B).

The prediction of putative NES and NLS signals suggested that LIF2 could be a shuttling protein between the nucleus and the cytoplasm. Therefore, we investigated the LIF2 subcellular localization by using translational fusions between LIF2 and the GFP marker in various systems (Fig. 2C–M). We firstly used transient expression assays in *N. benthamiana* and *A. thaliana* (Fig. 2C–E). Both the GFP:LIF2 and LIF2:GFP fusion proteins were targeted to the nucleus, indicating that LIF2 has a functional NLS. In *N. benthamiana*, a strong cytoplasmic signal was also detected with the LIF2:GFP construct, whereas a weak cytoplasmic signal was present in the cytoplasm of *A. thaliana* cells expressing transiently the GFP:LIF2 fusion protein (Fig. 2E). Similar results were obtained in transgenic *Arabidopsis* cell cultures (data not shown). Thus, LIF2 is targeted to the nucleus, but LIF2 protein can also be localized in the cytoplasm, possibly depending on the fusion orientation of the fluorescent marker, on the host type cells or the transformation technique.

To further test these hypotheses, the distribution of LIF2 was investigated in stable *Arabidopsis* transgenic plants. We analyzed several independent transgenic lines in both wild-type and *lif2-1* backgrounds (Fig. 2F–M). The two fusion proteins were targeted to the nucleus, in all the various tissues examined. Again, the LIF2:GFP fusion protein was found in both the cytoplasm and nucleus in all transgenic tissues indicating that the previous results were not due to the transient assay artifacts. Cytoplasmic fluorescence was also detected in lines expressing the GFP:LIF2 fusion protein construct, but with a weaker signal compared to the LIF2:GFP construct, indicating that the cytoplasmic localization is not strictly dependent on the fusion orientation. The intensity of this signal depended also on the tissue observed: fluorescence was more intense in the cytoplasm of guard and hypocotyl cells than in root cells (Fig. 2F–J). No « in foci » pattern resembling that described for LHP1 [22] was observed. A slightly heterogeneous nuclear distribution was observed in the root hair cells of GFP:LIF2 lines, with an exclusion from the nucleolus (Fig. 2L–M). These results showed that besides an expected nuclear localization as a partner of the LHP1 chromatin protein, a fraction of LIF2 molecules was consistently detected in the cytoplasm, suggesting that LIF2 shuttles between both compartments. The regulation of nuclear import/export of RNA-binding proteins (RBPs) may depend on various factors [51]–[55]. Further investigation will be required to determine the contributions of the LIF2 motifs and RNA partners in its subcellular distribution.

### Loss of function of *LIF2* causes a mild early flowering phenotype and a reduction of the rosette diameter

**Figure 3:**
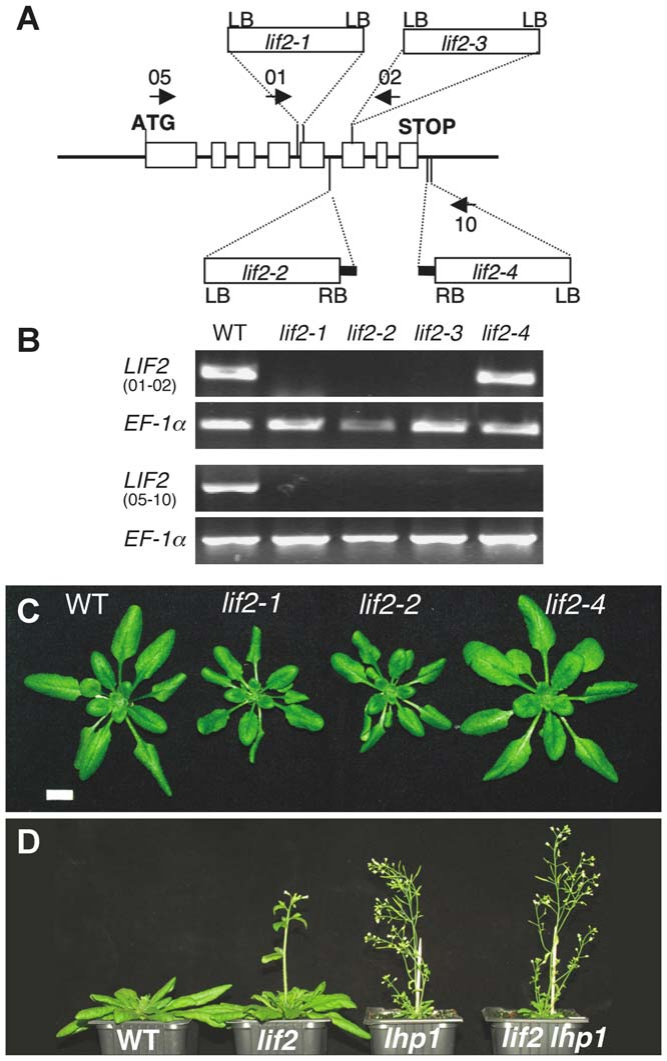
Characterization of *lif2* mutants. (A) Location of the *lif2* T-DNA insertions. Exons (open boxes). LB/RB: left and right borders of the T-DNA. Sequencing of the T-DNA flanking regions revealed small genomic deletions (*lif2-1*, *lif2-4*) or small genomic insertions (black boxes) (*lif2-2*, *lif2-4*). Arrows: Primers used. (B) *LIF2* expression in wild type (WT) and various *lif2* mutants. (C) Four-week-old WT and *lif2* plants under LD conditions. Scale bar: 1 cm. (D) Flowering phenotypes of five-week-old plants of WT and *lif2*, *lhp1* and *lif2 lhp1* mutants under LD conditions.

We studied the function of LIF2 by characterizing four T-DNA insertion lines, designated *lif2-1* to *lif2-4* and by analyzing their phenotypes (Fig. 3). The *lif2-1*, *lif2-2* and *lif2-3* alleles carry insertions in the coding sequence, while *lif2-4* has an insertion in the 3′-UTR (Fig.3A). RT-PCR analysis showed that *lif2-1*, *lif2-2* and *lif2-3* mutants have no LIF2 mRNA and are therefore likely null mutations (Fig. 3B). A *LIF2* transcript spanning the whole ORF could only be detected in the *lif2-4* mutant (Fig. 3B). As the T-DNA insertion is localized downstream of the Stop codon in the *lif2-4* mutant and its phenotype is similar to wild type, it is likely that LIF2 protein is produced. At vegetative stages, *lif2-1*, *lif2-2* and *lif2-3* rosettes had slightly downward-curled and smaller leaves compared to WT (Fig. 3C). Epidermal cell size was not affected in the *lif2* mutants (data not shown).

The length of the primary root in *lif2* mutants and wild type plants (9.8±0.6 and 8.2±1.2 cm in *lif2-1* and Col-0, respectively) and number of secondary roots (15.4±3.8 and 11.3±3.1 in *lif2-1* and Col-0, respectively) were not significantly different. A mild early flowering time phenotype was observed in the *lif2-1* to *lif2-3* mutants both in short-day (SD) and long-day (LD) conditions (Fig. 3D) (Table 1). However, this *lif2* mutant phenotype was not as strong as in *lhp1* mutants. In certain conditions, such as continuous light, at 15°C, *lif2* mutants and wild-type plants flowered with the same leaf number (total leaves 36.7±5.0 in Col-0 and 34.8±4.5 in *lif2-1*).

**Table 1:** Flowering time phenotypes of *lif2* mutants in short-day (SD) and long-day (LD) conditions.

	Rosette leaves		Total leaves		Flowering time(days)	Rosette diameter (cm)
	SD	LD	SD	LD	LD	LD
Col-0	71.6±7.3	21.1±6.0	81.3±7.0	25.6±6.8	41±5.3	7.6±1.3
*lif2-1*	63.7±4.7	13.4±0.9	75.7±4.5	17.1±1.1	32.8±1.7	7.5±0.8
*lif2-2*	57.5±2.4	11.7±1.6	68.2±2.7	14.4±1.7	32.0±1.8	5.7±2.2
*lif2-3*	60.4±2.4	12.7±1.2	73.4±2.6	15.4±1.1	32.1±2.6	6.1±0.9
*lif2-4*	64.5±1.6	14.0±1.7	73.7±1.7	17.7±2.3	31.0±2.3	9.9±1.3

SD conditions: 10 h/14 h (light/dark), 20°C/15°C (light/dark). LD conditions: 16 h/8 h (light/dark), 23°C/15°C (light/dark). Eight to 10 plants were analyzed for each genotype under each condition. Flowering time was recorded as the appearance of a 1 cm stem bolt. ± standard deviation. Rosette diameter represents the mean diameter from 8 to 10 plants.

We investigated the genetic interactions of *LIF2* with *LHP1*, by generating the *lif2-1 lhp1-6* double mutant. The *lif2-1 lhp1-6* mutant had a phenotype similar to that of *lhp1-6*, with a very small rosette, curly leaves and a reduced plant height (Fig. 3D). Both *lhp1* and *lif2 lhp1* mutants had similar flowering times, in terms of number of days till bolting (21.5±0.7 and 20.7±0.6, respectively versus 34±0.9 for *lif2-1*) and rosette leaf numbers (9±0.5 and 8.5±0.5, respectively, versus 20.8±1.5 for *lif2-1*). Furthermore, the *lhp1* and *lif2 lhp1* mutants showed a similar mean number of branches per rosette (7.5±1.1 and 7.5±0.9, respectively) or number of secondary inflorescences per branch (3.2±0.3 and 3.3±0.2, respectively).

Flowering shoots (inflorescences) of wild-type *A. thaliana* grow indeterminately, maintaining shoot inflorescence meristem identity until they finally senescence. In *lhp1/tfl2* mutants, inflorescence meristems are converted to floral meristems thus switching from indeterminate-to-determinate inflorescences producing terminal flowers [29], [56]. Similarly, the termination of inflorescence growth was observed in the *lif2 lhp1* mutant, which developed terminal flowers whereas the *lif2* floral abnormalities in the first flowers of the main inflorescence were not observed. Overall, our results suggest that LIF2 acts mainly downstream of LHP1.

### LIF2 regulates flower development

**Figure 4:**
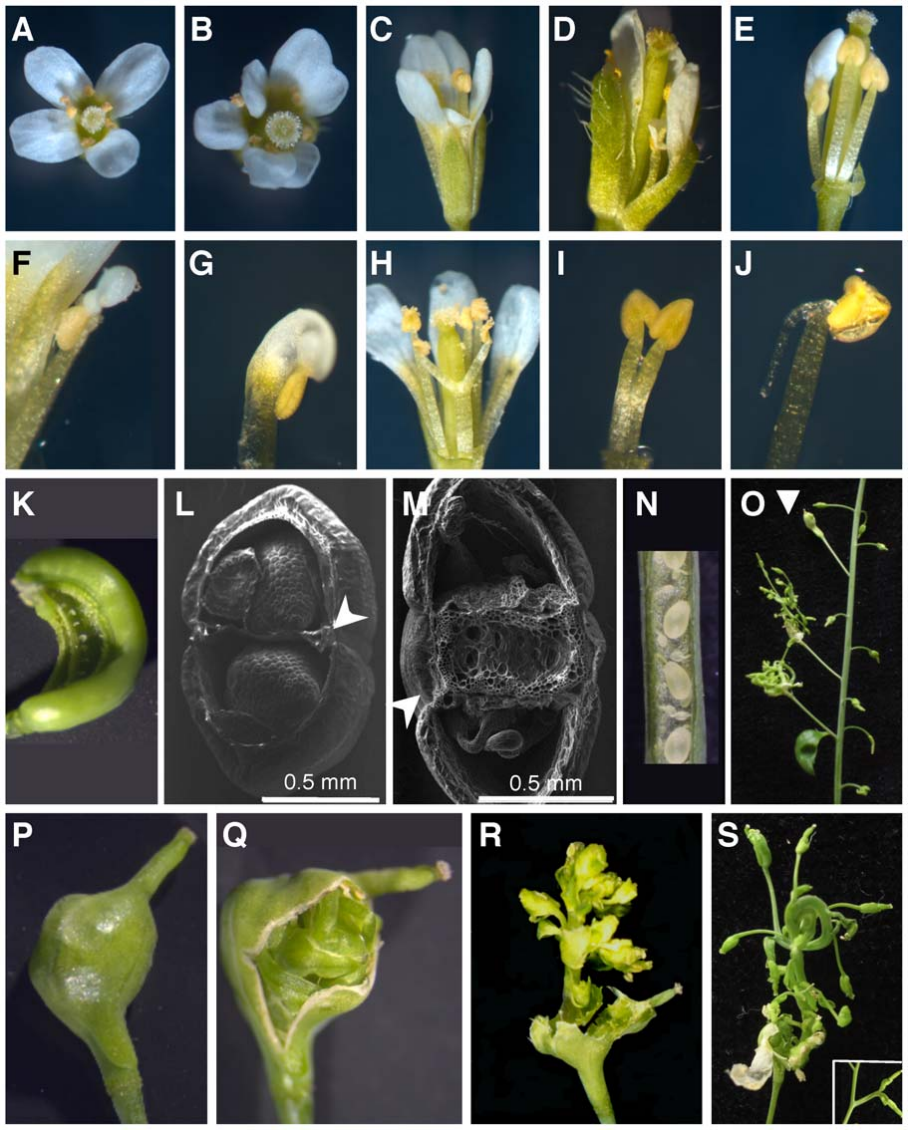
Abnormal flower development in *lif2* mutants. (A) A *lif2-1* flower with a phenotype similar to wild-type. (B–E) *lif2-1* flowers with abnormal phenotypes. (F) Staminoid petal, (G) petaloid anther, (H, I), twin anthers and (J) anther with a spur-like structure. (K) Abnormal silique with an open carpel and visible ovules. (L–M) SEM photographs on transversal sections of *lif2* siliques with (L) normal and (M) enlarged replum (arrows). (N) Ovule abortion in a *lif2* silique. (O–S) Indeterminate ovary (IDO) phenotypes. Primary inflorescence of a *lif2* mutant grown in SD conditions, bearing a closed IDO (arrow) and below, two IDOs with visible ectopic inflorescences erupting from them. (P–S) Close views of (P) a closed IDO, (Q) an IDO with the ectopic inflorescence starting to emerge, and (R) an IDO with its ectopic inflorescence emerging. (S) Ectopic IDO inflorescence showing stem fasciation and flowers at various developmental stages. Closer view of fusion between floral pedicels.

We further investigated flower development in *lif2* mutants and observed several defects in *lif2-1*, *lif2-2* and *lif2-3* mutants (Fig. 4), but none in *lif2-4*. In LD conditions the developmental abnormalities were particularly prevalent in the first 50 flowers of the main inflorescence stem and affected the first three floral whorls with different degrees (Fig. 4A–D). By contrast, upper flowers had WT phenotype. In the first abnormal flowers, organ number varied from four to seven in the first whorl and from two to five in the second whorl (Table 2). The most common defect was the absence of one or two stamens in the third whorl (Table 2). Some flowers showed fusions of organs and chimeric organs with partial homeotic conversions (Table 2). Sectors of sepal were transformed into petals or petals into staminoid organs. Petaloid tissues at the tip of the anthers (Fig. 4E–G) or twin anthers were also observed, with fusions at various locations along the filaments (Fig. 4H–I) and the formation of extra tissue (spur-like) at the junction between anther and filament (Fig. 4J). Abnormal siliques with a deformed style, enlarged replum or one open carpel with visible young ovules were also observed (Fig. 4K–M).

**Table 2:** Floral phenotypes of *lif2* mutants.

Primary Inflorescence (%)	Col-0	*lif2-1*	*lif2-3*
4S	94.6	71.6	75.0
5S	4.9	21.6	21.2
6S	0.4	6.1	3.3
7S	0.0	0.7	0.5
2P	0.0	1.4	0.5
3P	0.0	14.2	13.6
4P	99.6	82.4	84.2
5P	0.0	2.0	2.2
6P	0.4	0.0	0.0
446	61.2	10.1	27.7
445	27.7	37.2	24.5
444	4.5	10.8	12.0
Abnormal	6.7	41.9	35.9
Petaloid sepal	0.0	1.4	0.5
Staminoid petal	0.0	10.8	14.7
Petaloid stamen	0.0	0.0	1.1
Twin anthers	0.4	1.4	1.1

Floral organs were counted in a total of 120 to 180 first flowers from 6 primary inflorescences in LD1 conditions (16 h/8 h, 18°C/15°C (light/dark)). xS or xP: flowers with x organs (S, sepal; P, petal). xyz phenotype: x sepals, y petals, z stamens. Flowers with “abnormal” phenotypes have xyz phenotypes different from the three main classes, showing twin stamens or chimeric organs. Numbers correspond to the % of total flowers analyzed.

We further investigated whether growth conditions or the position of the flowers on the inflorescence could enhance flower phenotypes. We observed that flowers on the primary inflorescence were more affected than flowers on the secondary inflorescences and the proportion of abnormalities was higher in SD conditions (Table 3). The *lif2* floral phenotype is thus variable along the axis of the inflorescence stem and depends on environmental factors. Finally, the *lif2* flowers were less fertile than the wild type, with smaller siliques in LD and SD conditions, this phenotype being slightly more pronounced in SD and continuous light conditions. Ovule abortion seemed to contribute to this reduced fertility (Fig. 4N).

**Table 3:** Floral *lif2* phenotypes in different growth conditions.

Phenotype 446(in %)	SD	LD1	LD2	LD3	CL1
Col-0	87.6 (nd)	61.2 (68.1)	66.4 (nd)	73.7 (nd)	21.6 (53.3)
*lif2-1*	28.2 (47.3)	10.1 (32.6)	22.5 (nd)	27.6 (nd)	19.3 (8.1)

Phenotype 445					
Col-0	8.2 (nd)	27.7 (20.6)	22.9 (nd)	20.2 (nd)	36.3 (31.3)
*lif2-1*	15.3 (40)	37.2 (18)	39.1 (nd)	34.6 (nd)	31.3 (28.6)

Phenotype 444					
Col-0	1 (nd)	4.5 (7.5)	6.9 (nd)	1.2 (nd)	32.6 (6)
*lif2-1*	1.2 (9.1)	10.8 (5.3)	18.8 (nd)	13.4 (nd)	22.9 (18.6)

Abnormal					
Col-0	3.1 (nd)	6.7 (3.8)	3.8 (nd)	4.9 (nd)	9.5 (9.3)
*lif2-1*	55.2 (3.6)	41.9 (44.4)	19.6 (nd)	24.4 (nd)	26.6 (44.7)

Floral organs were analyzed on a similar number of plants and flowers as in Table 2. Numbers correspond to the % of total flowers analyzed. The primary and the first secondary (in brackets) inflorescences were analyzed. Growth conditions: SD: 14 h/10 h (light/dark), 20°C/15°C (light/dark); LD1 (Table 2); LD2: 16 h/8 h (light/dark), 23°C/15°C (light/dark); LD3: LD 16 h/8 h (light/dark), 20°C; CL1: continuous light, 15°C.

### LIF2 maintains ovary determinacy in SD conditions

Unlike the inflorescence meristem, the wild-type floral meristem is determinate: it is eventually consumed in the production of four whorls of floral organs, which terminate its development [57]. In SD conditions, swollen gynoecia were observed in the first ten flowers of the *lif2* primary inflorescence, and the central regions of these floral meristems produced ectopic inflorescences (Fig. 4O–S). These structures were named indeterminate ovaries (IDO) due to their growth pattern (Fig. 4P–Q). The *lif2* IDO ectopic inflorescences displayed fasciation of stem, fusion of pedicels of the flowers, abnormal flowers with organ fusions, and changes in organ number and identity (Fig. 4R–S).

**Figure 5:**
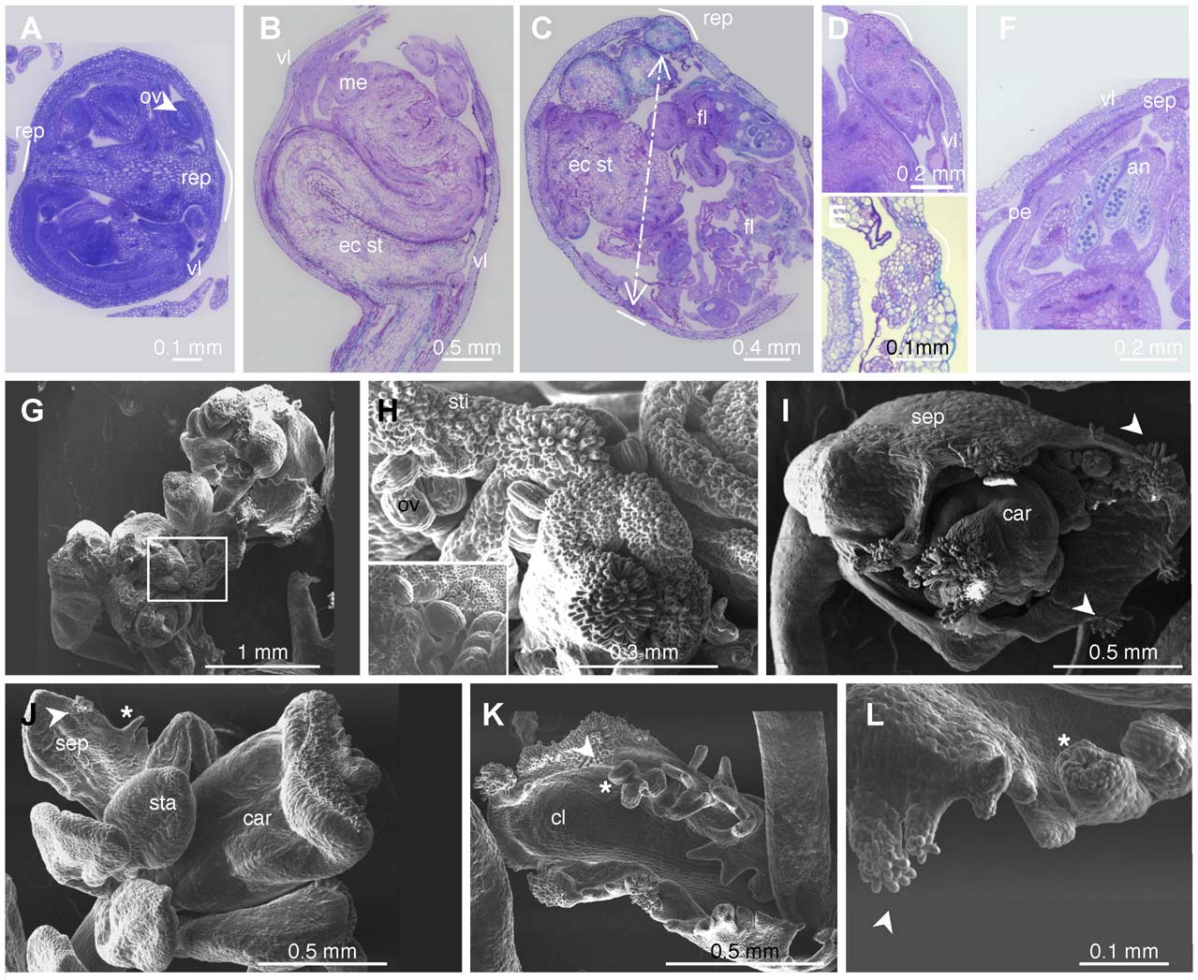
Structure and organization of the *lif2* indeterminate ovary (IDO). (A–F) Toluidine Blue O stained sections of (A) young and (B–F) older IDOs. Arrow in C indicates the two replum regions. (G–L) SEM photographs of ectopic inflorescence and flowers from IDOs. (G) Young ectopic IDO inflorescence. (H) Closer view of the selected square region in G showing proliferation of stigmatic papillae and ovules. (I) Abnormal floral bud with proliferation of ectopic stigmatic papillae (arrows). (J) Abnormal flower from an IDO with serrate sepal (star). (K) Cauline leaf with ovules (star) and stigmatic papillae. (L) Closer view of the margin of a cauline leaf with proliferating tissues with stigmatic papillae and ovule-like structures. sti: stigmatic papillae. ov: ovule. sep: sepal. sta: stamen. car: carpel. cl: cauline leaf.

Light and electron microscopy analyses were conducted to better describe the IDO phenotype (Fig. 5). In wild-type flowers, the replum develops symmetrically, but early in the IDO development on the *lif2* mutant, an asymmetrical growth of the replum region was observed (Fig. 5A). The asymmetric growth of the replum seemed to parallel the development of the ectopic inflorescence, which occupied most of the volume of the gynoecium, the tissues located between the two valves being disrupted in late IDO development stages before the final asymmetric disruption of the gynoecium (Fig. 5B–E). The ectopic inflorescence meristem produced abnormal flowers (Fig. 5F–J) and cauline leaves, with proliferations of stigmatic papilla and ovules (Fig. 5H–I, K–L). Furthermore, the surface of the organs exhibited various cell types suggesting perturbations of cell proliferation and cell identity (Fig. 5J). Despite these severe abnormalities, a few viable seeds were made by IDOs, which produced plants with *lif2* phenotypes, but without any enhancement of the IDO phenotype. These results suggest that *lif2* mutation induces various flower developmental defects and indeterminate growth of the ovary in response to environmental perturbations.

### LIF2 modifies the expression of a subset of LHP1 target genes

**Figure 6:**
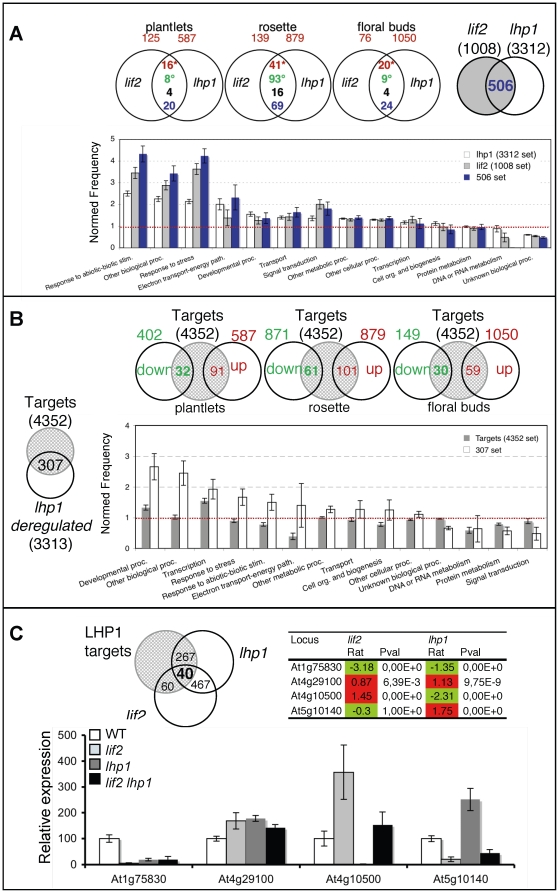
Analyses and comparisons of *lif2* and *lhp1* transcriptome profiles with LHP1 genomic distribution. (A) *lhp1* and *lif2* transcriptome comparisons in young seedlings, rosette leaves and floral buds. Venn diagrams were generated (http://www.pangloss.com/seidel/Protocols/venn.cgi) indicating the numbers of deregulated genes: upregulated in red; downregulated in green; up in *lif2*/down in *lhp1* in black; down in *lif2*/up in *lhp1* in blue (upper panel). Gene ontology data (GO) of the three gene sets corresponding to genes deregulated in *lif2* (1008), *lhp1* (3312) and in both mutants (506) were extracted. A normed frequency was calculated which represents the frequency normalized to the number of genes in each GO class in the genome by using the BAR superviewer program (http://bar.utoronto.ca/ntools/cgi-bin/ntools_classification_superviewer.cgi) and the histogram of the values was produced to highlight relative enrichments of the GO classes (lower panel). (B) Comparison between the *lhp1* transcriptome data (this study) and 4352 LHP1 targets [20]. Numbers of LHP1 targets deregulated (up or down) in each experiment are indicated. A set of 307 LHP1-bound and *lhp1*-regulated loci (“Lbr” genes) was identified among the 3313 deregulated genes suggesting a specific requirement of LHP1 for their regulation (upper panel). The normed frequencies of the GO classes for the 4352 LHP1 targets (grey) and the 307-Lbr set (white) were calculated using BAR superviewer and the histogram of the values is presented. (C) A 40-gene set corresponding to genes both targeted by LHP1 and deregulated in both *lif2* and *lhp1* was extracted from lists established in A and B. Selected genes among the 40-set for their representative expression patterns in *lif2* and *lhp1* based on CATMA data: the log ratio (Rat) of the microarray fluorescence signals and P-values are indicated. The mRNA levels of 4 selected genes (indicated) were detected by qRT-PCR analysis in WT and mutant rosette leaves after bolting. The mRNA levels (relative to *EF1α* transcript level) in wild type were set as 100. Data in the graphs are the average of at least three qRT-PCR assays from two independent experiments; the bars represent standard error.

Despite *in vivo* interaction of LIF2 with LHP1 in the nucleus of plant cells, *lif2* and *lhp1* phenotypes did not show many common characteristics. Therefore, we investigated at the molecular level if some genes were commonly deregulated in both mutants. Both *lif2* and *lhp1* transcriptome profiles were determined by using Complete Arabidopsis Transcriptome MicroArrays (CATMAs) [58], [59]. Transcriptome profiles were performed in *in vitro* plantlets, rosette leaves and floral buds (Fig. 6). Gene expression was globally more strongly affected in *lhp1* than in *lif2*. Among the 21643 nuclear genes present on the CATMA array, 3312 were deregulated in at least one condition in *lhp1*, whereas 1008 were deregulated in *lif2* (the lists of genes were established by adding deregulated genes in the different biological materials and by removing duplicates) (Fig. 6A). The numbers of genes up or down regulated were similar for a particular mutant in the different conditions except for two conditions: 80.9% of the deregulated genes in *lif2* rosette were down regulated, whereas 87.6% were up regulated in *lhp1* floral buds, which reflect an overall global tendency (i.e. 72.7% down-regulated in *lif2* compared to 67% up-regulated genes in *lhp1*). These data suggest that LIF2 has a smaller impact than LHP1 on the transcriptional regulation of the whole genome. Furthermore, *LHP1* seems more involved in gene repression globally, as expected for a Polycomb subunit, whereas *LIF2* has a more general gene activation effect. However, despite these global antagonistic effects, 50% to 61% of the deregulated genes in both mutants were deregulated in the same way, in the same tissues, suggesting also common regulation pathways.

To better understand these pathways, we analyzed the Gene Ontology (GO) of the deregulated gene sets using the Bio-Array Resource for Plant Functional Genomics (BAR) classification superviewer program [60] (Fig. 6A). The 1008-set of *lif2-*deregulated genes showed a strong GO enrichment for genes involved in responses to abiotic and biotic stress stimuli, whereas this enrichment in *lhp1* was weaker (Fig. 6A). Interestingly, for the 506-set corresponding to genes deregulated in both mutants, we observed a higher enrichment in responses to abiotic and biotic stress stimuli compared to the two mutants sets (Fig. 6A).

We next wondered whether the two proteins could act on the same LHP1 target genes. In a previous study, we identified 4352 regions physically-bound by LHP1 by the DNA adenine methyltransferase identification (DamID) method, coupled with microarray hybridization for a genome wide identification [20], [23]. We firstly compared *lhp1* transcriptome profiles to the 4352-LHP1 target set. We identified 307 LHP1-bound and *lhp1*-regulated loci (“Lbr” genes). The 307-set represents a small fraction of the LHP1 targets (7%) specifically regulated by LHP1 directly, suggesting either that the material used for transcriptome analysis could not reveal these other LHP1 targets, or redundancy with other chromatin regulators and PRC components to regulate the majority of the LHP1 targets. Interestingly, no particular GO category enrichment was detected among the LHP1-bound loci, but we observed a significant enrichment in genes associated with developmental processes, transcription and responses to stress in the 307-Lbr gene set suggesting that these genes are mainly regulated by *LHP1* (Fig. 6B). Among the 21 Lbr transcription factors identified in the 307-Lbr set, half of them belong to the MADS family, some being already described (i.e. *AGAMOUS* (*AG*), *PISTILLATA* (*PI*), *FLOWERING LOCUS C* (*FLC*)) [23]. New MADS box-LHP1 target genes were also identified such as *SEPALLATA* (*SEP1* to *SEP4*), *SHATTERPROOF1* (*SHP1*) and *SHP2*, which are involved in floral or ovule development (GO:0048440 carpel development, p-value: 2.5 10^-09^, EasyGO).

We finally compared the 307-Lbr set to the *lif2* transcriptome data and identified 40 genes which are LHP1-bound and deregulated in both *lif2* and *lhp1* mutants. The GO assignment of these genes did not reveal any major enrichment in molecular functions, but did show a relative enrichment in responses to environmental cues (data not shown). Among these 40 genes, no enrichment in up or down regulated genes in the two mutants could be revealed. We chose four genes (At1g75830, At4g29100, At4g10500, At5g10140) with various expression patterns in *lif2* and *lhp1* mutants (Fig. 6C). Their expression was monitored by quantitative RT-PCR (qRT-PCR) and this confirmed the CATMA data (Fig. 6C). Our transcriptome analysis coupled with the identification of LHP1 targets revealed that *LIF2* and *LHP1* regulate a common small set of genes, most of them being involved in responses of various environmental cues.

### LHP1 binding appears independent of LIF2

Interaction between *LIF2* and *LHP1* was further studied in the *lif2 lhp1* mutant by analyzing the expression of the four previously selected genes. Interestingly, the expression of two genes (At1g75830, At4g29100), which had similar expression patterns in both mutants, did not change significantly in *lif2 lhp1*. In contrast, there was a restoration to a wild-type like expression level for the two other genes (At4g10500, At5g10140) which had opposite expression patterns in single mutants (Fig. 6C). Overall, these analyses suggest that LHP1 and LIF2 could act on a subset of LHP1 target loci either antagonistically or agonistically.

**Figure 7:**
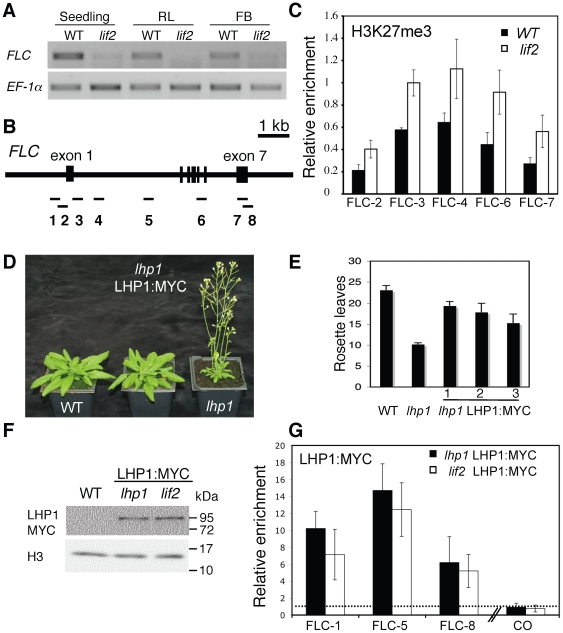
H3K27me3 and LHP1 distribution on *FLC.* (A) Expression at various developmental stages in wild-type and *lif2* plants. Semi-quantitative RT-PCR were performed on seven-day-old *in vitro* seedlings, rosette leaves after bolting (RL), floral buds just after bolting (FB1) and floral buds after the production of 10 siliques (FB2). The *EF-1α* gene expression was used as a control. (B) Schematic representation of the *FLC* locus and the 8 amplified regions used in chromatin immunoprecipitation (ChIP) assays. (C) ChIP analysis to determine the relative level of H3K27me3 at the indicated *FLC* regions in wild-type and *lif2* seedlings. Immunoprecipitated DNA was analyzed by real-time qPCR, and enrichment was calculated as percentage of INPUT. All ChIP experiments were normalized for histone H3 occupancy and normed by using ChIP results on an *AGAMOUS* control region. Data in the graphs are the average of at least two qPCR assays from three independent ChIP experiments; the bars represent standard error. (D–F) Complementation of the *lhp1* mutant by the expression of the genomic LHP1:MYC-tagged construct. (D) Plant phenotypes, (E) total number of rosette leaves, and (F) protein levels. (G) ChIP assays to determine the relative level of LHP1 binding at the indicated *FLC* regions in wild-type and *lif2* backgrounds expressing the LHP1:MYC-tagged construct. A *CONSTANS* (CO) region was used as a negative control [23]. Immunoprecipitated DNA was analyzed by real-time qPCR, and enrichment was calculated as percentage of INPUT and normalized relative to a Col-0 control, represented as a dashed line. Data in the graphs are the average of at least two qPCR assays from three independent ChIP experiments; the bars represent standard error.

We next wondered whether the antagonistic effect was associated with histone post-translational modifications and/or changes in LHP1 binding. We used At5g10140, encoding the MADS box transcriptional repressor of floral transition *FLC*, as a well-studied example. We first checked *FLC* expression at various developmental stages in *lif2* mutants and could observed a consistent down-regulation (Fig. 7A), which was associated with an increase in H3K27 trimethylation (Fig. 7B–C). To investigate LHP1 binding in the *lif2* mutant, we generated a genomic LHP1:MYC construct; this genomic fragment had been previously used for *lhp1* complementation [26]. As expected, the genomic LHP1:MYC construct could restore a WT phenotype when introduced into the *lhp1* mutant (Fig. 7D–E) and a LHP1:MYC fusion protein could be easily detected (Fig. 7F). Using Myc-epitope tagged LHP1 transgenic plants, no major change in LHP1 binding could be detected by chromatin immunoprecipitation (ChIP) on the *FLC* locus despite its change in expression in *lif2* mutant (Fig. 7G).

## Discussion

In eukaryotes, proteins of the HETEROCHROMATIN PROTEIN1 family are characterized by two conserved domains, the chromo domain and the chromo shadow domain. These domains allow interactions with numerous proteins whose functions are highly diverse and which confer a platform function to the HP1 protein family [61], [62]. In this study, we identified LHP1-INTERACTING FACTOR2, a new partner of LHP1, the plant structurally-related HP1 protein. We showed that the LHP1-LIF2 interaction was mediated by the conserved chromo shadow domain of LHP1 and occurred *in vivo* in the plant cell nucleus. Our localization data showed that LIF2 is a nucleocytoplasmic protein, suggesting that besides functioning in chromatin dynamics and LHP1 regulation, LIF2 has additional functions.

LIF2 contains three RNA-recognition motifs (RRM) suggesting that LIF2 may bind to single-stranded RNA molecules, whose nature remains to be determined. However, we can not exclude interaction with other nucleic acids or proteins since some versatile RRM functions have also been described [41], [42], [63]. Plants have developed a larger and more complex set of RRM-containing proteins than animals [64], [65], suggesting the existence of plant-specific RNA processing mechanisms which remain to be explored. Only a few functional studies have been reported on plant RRM-containing proteins [65]–[71]. By showing that *LIF2* is involved in promoting expression of the key floral repressor *FLC*, we have added another component to the growing list of RRM-containing proteins and RBPs (i.e. FCA, FPA, FY, AtGRP7, FLK and PEPPER) which either promote or repress *FLC*, tightly regulated by both epigenetic modifications and an RNA processing mechanism [66], [72]–[81].

Based on LIF2 similarity with other eukaryotic RRM-containing proteins, LIF2 belongs to the hnRNP large protein family, which includes RBPs involved in various functions, ranging from transcriptional to post-transcriptional regulation and RNA processing. At the time of its identification, no RNA binding protein was identified as a partner of the HP1 protein family. Since then, however, recent studies have shown that the hnRNP U protein physically interacts with HP1*α* [82] and that hnRNP U/SAF-A associates with HP1γ in the nuclear compartment [83], [84]. This reinforces our original choice to investigate the LHP1/LIF2 interaction. Thus, the interaction between HP1/LHP1 proteins and hnRNP proteins seems to be a common theme both in plants and animals.

### LIF2 controls cell identity during flower development and gynoecium determinacy

Floral meristems contain a transient pool of stem cells that produce a determinate number of floral organs before terminating their activity during carpel formation. In this study, we showed that LIF2 is a floral development regulator, controlling the number and identity of floral organs, and that it is a regulator of floral determinacy by maintaining a determinate growth of the gynoecium. A strong developmental reversion from floral to inflorescence development has been observed in basal *lif2* ovaries in SD conditions. This is in some respects opposite to the terminal flower phenotype of the *lhp1* mutant whose main inflorescence meristem is consumed by floral formation. The ectopic inflorescences showed fasciation and carried abnormal flowers with proliferation of papilla, a phenotype also observed in *Atring1a Atring1b* flowers [30]. Reporter lines expressing GUS under the *LIF2* promoter revealed expression in the gynoecium and the gynophore, in agreement with the IDO phenotype.

Few reversions of floral meristem commitment have been reported in *A. thaliana* [85], [86]. Such unusual events have been observed in transgenic *Arabidopsis* lines expressing constitutively the *AGL24* MADS-box flowering transcription factor [87]; in *lfy6+/−* or *ag1−/−* flowers in SD conditions [88]; in *ult1 clv1-4* gynoecia [89]; or in early flowers of *Sy-0 Arabidopsis* plants [90], [91]. All these examples displayed floral transformations with production of ectopic inflorescence shoot meristem. The *lif2* IDO phenotype is also reminiscent of the *crc rbl*, *crc sqn* or *crc ult1* double mutant phenotypes showing ectopic floral organs developing inside carpels [92]. Thus, similarly to *REBELOTE* (*RBL*) and *SQUINT* (*SQN*), *LIF2* may control floral developmental homeostasis. The patterning and maintenance of meristematic cells in the gynoecium are driven by complex mechanisms not clearly understood. Parallels have been drawn between the formation of shoots and ovules [93], suggesting the possible reorientation of the cell fate of carpel margin meristems or ovule primordia towards the formation of a new inflorescence meristem. The IDOs also showed abnormal replum growth. Whether this tissue might also participate to such reversion remains to be established and further analyses are required to identify the origin of the new inflorescence meristem in the *lif2* indeterminate ovary.

In SD conditions, the frequency of floral developmental abnormalities gradually declined towards the top of the inflorescence. These findings suggest that the amount, activity or perception of signals involved in floral determinacy may vary with time or distance during inflorescence development and with environmental conditions. In wild-type plants, there may be a mechanism ensuring robust floral determinacy and patterning that involves LIF2, particularly in the first flowers, in SD conditions. This mechanism may become less important as development proceeds, with the floral determinacy being more strongly established by other reinforcing mechanisms. The possible redundancy of related *LIF2* genes may also contribute to the transient aspect of some of the observed phenotypes.

### *LIF2* regulates LHP1 target genes by possibly modulating LHP1 activity

We could show that despite sharing only a few phenotypic traits, such as early flowering time and reduced rosette size, *LHP1* and *LIF2* have overlapping molecular function. Indeed, a set of 506 genes deregulated in both mutants could be identified. Interestingly, this 506-set showed a high enrichment in genes involved in biotic and abiotic stress responses, whereas no bias could be identified among the target genes physically-bound by LHP1, as identified by the DamID technique [20]. Enrichment in genes involved in developmental processes was observed in deregulated genes in the *lhp1* mutant in agreement with the pleiotropic *lhp1* mutant phenotype. These data suggest a combined role for *LIF2* and *LHP1* to regulate genes in responses to various environmental cues. Transcriptome data in the *lhp1* mutant combined with LHP1 chromatin profiling showed that in two out of three conditions (seedling and rosette) the same numbers of LHP1 targets genes were up and down regulated. This is in agreement with the wide role of HP1 proteins in gene repression or activation and thus acting as modulators of gene transcription [94]. A similar function for LHP1 is thus expected.

By investigating the regulation of four LHP1 target genes, we could show that LHP1 and LIF2 can act antagonistically on gene expression. How both LHP1 and LIF2 converge to regulate gene expression remains to be further explored. However, we could demonstrate by monitoring LHP1 binding in the *lif2* mutant that LIF2 is not involved in LHP1 targeting at the *FLC* locus. Also, LIF2 can influence histone post-translational modifications, such as H3K27 trimethylation, as this mark increased at a silenced LHP1 target gene in the *lif2* mutant. We showed that LHP1 binding can be associated with a silent (i.e. in *lif2* mutant) or an active (i.e. in WT) transcriptional status of *FLC* locus suggesting that LIF2 and most probably other components can modulate LHP1 activity.

### Some emerging links between RNA processing and Polycomb regulation in plants

Evidence is accumulating to suggest that RNA components play a key role in chromatin dynamics and gene regulation. Indeed RNA is involved in the establishment of chromatin marks via an RNA-directed DNA methylation pathway [90], [95]–[98]. A loss of function in components of the RNA interference machinery in *S. pombe*, *Drosophila* and mouse results in an abnormal distribution of HP1 and defects in heterochromatin formation [99]–[105]. Also, HP1*α* interacts with PIWI protein, an ARGONAUTE/PIWI family protein interacting with non-coding RNA involved in silencing [106]. HP1 chromatin complexes have also been shown to be involved in crosstalk between the transcriptional machinery, RNA processing and chromatin dynamics [107]–[113]. Various links between PcG silencing and RNA components have also been demonstrated in animals [8], [114]–[120]. Thus, the identification of the RNA binding protein LIF2, as a partner of LHP1, a functional subunit of a plant PRC1-like complex, opens up new perspectives in gene regulation by plant chromatin and provides novel links between Polycomb regulation and RNA processing to investigate. It also highlights LHP1 as an intriguing plant protein, at the interface between HP1 and PcG regulation, possibly contributing to plant plasticity.

## Materials and Methods

### Plant materials

All the *Arabidopsis thaliana* lines in this study were in the Columbia (Col-0) accession. The T-DNA insertion lines (SALK_021829, SALK_022139, SALK_021077 and SALK_062462) were obtained from the *Arabidopsis* Biological Resource Center (ABRC). Corresponding homozygous lines were named *lif2-1*, *lif2-2*, *lif2-3* and *lif2-4*, respectively. The *lhp1-4* (*tfl2-2*, CS3797 [40]) and the SALK_011762 line (named *lhp1-6* [121]) were also supplied by ABRC. For phenotypic analyses, plants were grown on soil in growth chambers, under controlled conditions as described previously [26]. *Nicotiana benthamiana* plants were grown in the greenhouse.

### Primers

All primers are described in Table S1.

### Yeast two-hybrid screening

The CD4-22 *Arabidopsis* WT (Col-0) λACT cDNA library (3×10^6^ independent clones) obtained from three-day-old etiolated seedlings [122] was used for yeast two-hybrid screening. The yeast strain PJ69-4A [123] expressing *LHP1* in frame with the sequence encoding the GAL4 DNA binding domain was transformed with the λACT cDNA library. Several transformations were performed with efficiencies ranging from 1×10^4^ to 1.7×10^5^ cfu/µg. In total, 2×10^6^ transformants were screened. Colonies were picked and re-streaked on selective media lacking histidine, leucine and tryptophan (-HLW), supplemented with 2 mM 3-amino-1,2,4-triazole. β-galactosidase assays were conducted on 380 selected clones. Plasmids were recovered from positive yeast clones and used subsequently to confirm interactions by independent cotransformations. Out of the 380 initial colonies, we sequenced 108 positive clones, which corresponded to 37 different genomic loci.

### Plasmid constructs

For pull-down experiments, a PCR fragment corresponding to the full-length *LIF2* coding sequence was amplified with the AD379c1 and AD379c2 primers using pda01769 as a template (Riken reference RAFL09-11-B19) [124]. The *LIF2* PCR fragment was digested with *Nco*I and inserted into the *Nco*I-digested pGEX4T2-*Nco*I plasmid derived from pGEX4T2 (Amersham) and harboring the glutathione S transferase (GST) tag. An *Nco*I fragment bearing the *LHP1* cDNA fragment [26] was inserted into the *NcoI* site of pET29a vector (Novagen) and the *Sac*I/*Xho*I adaptor was inserted between the *Sac*I and *Xho*I sites of the resulting plasmid to obtain an in-frame LHP1:His-tagged fusion. The LHP1 *Nco*I fragment was also inserted into the *Nco*I-digested pGEX4T2-*Nco*I plasmid. These steps generated the pGEX-LIF2, pET-LHP1 and pGEX-LHP1 plasmids.

For BiFC experiments, the *LIF2* and *LHP1* cDNA fragments were amplified by PCR with the AD379c1/AD379c2 primers and the NtermL2/CtermL2 primers, respectively. The PCR fragments were inserted into the *Bam*HI restriction site for the *LIF2* fragment, and between the *Xba*I and *Xho*I restriction sites for the *LHP1* fragment, in the pSPYNE-35S and pSPYCE-35S vectors [50] harboring the YFP N-terminal and YFP C-terminal fragments, respectively. The pSPYNE-LIF2, pSPYCE-LIF2, pSPYNE-LHP1 and pSPYCE-LHP1 plasmids were generated.

For the generation of transgenic lines producing the LIF2:GFP protein, the *LIF2* AD379c1/c2 PCR fragment was digested with *Nco*I or *Bam*HI, and inserted into the *Nco*I or *Bg*lII restriction sites of the pAVA121 vector encoding the S65T GFP protein under the control of the 35S CaMV promoter, to obtain GFP fused to the N-terminal and C-terminal ends of LIF2, respectively [26]. The P35S::LIF2:GFP and P35S::GFP:LIF2 constructs were then introduced into the pCambia1300 binary vector, generating the binary pCaLIF2:GFP and pCaGFP:LIF2 plasmids.

For *LIF2* expression analyses, a 3-kb-long *LIF2* promoter region was amplified from BAC plasmid T18A10 using primers AD379-28 and AD379-29 and inserted as a *Pst*I/blunt-ended fragment at the *Pst*I and *Hind*III blunt made sites of a pCambia1300 vector carrying the Nos terminator (pCa2LIF2 plasmid). The *uid*A gene encoding the β-glucuronidase (GUS) was amplified from the pBIG-KAN vector [125] using primers GUS-Pst and GUS-STOP and cloned into the *Pst*I/*Sma*I digested pCa2LIF2 plasmid.

For LHP1 profiling, the 5569 bp genomic LHP1 fragment of the pCaSSP binary vector was shown to fully complement the *lhp1-1* mutant [26]. The *Nco*I/*BstE*II fragment of pCaSSP was subcloned into pSK+ vector and mutagenized to replace the Stop codon by an *EcoR*V restriction site, in which a 10-Myc tag fragment, PCR amplified from the PGW19 vector (Invitrogen), was inserted. The *Nco*I/*Bst*EII fragment bearing the MYC tag was then substituted to the wild-type genomic fragment of the pCaSSP vector giving the LHP1:MYC binary plasmid.

### Pull-down assays

*E. coli* cells (Rosetta, Novagen) harboring various expression constructs were cultured at 37°C in 25 ml LB medium supplemented with appropriate antibiotics to obtain an OD_600_ of 0.6. The cultures were supplemented with 1 mM IPTG and transferred to 28°C, for 4 h. After centrifugation, bacterial pellets were resuspended in 2 ml of buffer (40 mM Tris-HCl, pH 7.4, 50 mM NaCl, 1 mM EDTA, 1 mM DTT) and lysozyme was added to 50 µg/ml on ice for 30 min, followed by two cycles of freezing and thawing. After centrifugation at 20,000 g for 30 min, at 4°C, supernatants were recovered and adjusted to obtain a final BB binding buffer composition [16]. Extracts containing GST- and His_6_-epitope tagged proteins were mixed in a 1∶2 ratio with equilibrated glutathione Sepharose 4B beads (Amersham) and incubated at room temperature for 1 h with gentle shaking. Beads were washed 5 times with binding buffer. After SDS-PAGE electrophoresis and electro-transfer onto nitrocellulose membrane, His_6_-epitope tagged proteins were detected with the anti-His_6_-peroxidase antibody (Roche) and the Immun-Star horseradish peroxidase chemiluminescence kit (Bio-Rad) according to the manufacturer's instructions. Assays were performed in duplicate.

### Transient assays

BiFC experiments were performed in *Nicotiana benthamiana* leaves as previously described [126]. For coinfiltration, each *Agrobacterium* strain was resuspended at an OD_600_ of 0.7 and mixed to a 1∶1 ratio. Leaves were infiltrated using a 5 mL syringe and observed 48 to 72 h after infiltration. *In planta* transient transformation assays were performed in young *Arabidopsis* seedlings and fluorescence was recorded 3 days later by confocal microscopy [127].

### Expression analysis

Total RNA was isolated from various tissues, using the RNeasy Plant Mini Kit (QIAGEN) and treated with RNase-free DNaseI (Invitrogen). Reverse transcription (RT) reactions were performed with Superscript II reverse transcriptase (Invitrogen) according to manufacturer's instructions. Quantitative real-time PCR (qPCR) were performed on Eppendorf Mastercycler® ep realplex (Eppendorf) using MESA FAST qPCR MasterMix Plus for SYBR® Assay (Eurogentec) as manufacturer's instructions.

GUS histochemical staining analyses were performed in the T2 generations of seven transgenic lines as described [128].

### Transcriptome analyses

Transcriptome analyses were performed on *lif2-1* and *lhp1-2* mutants using CATMA arrays [58], [59], [129]. *In vitro* plantlets were grown on basal salt Murashige and Skoog (MS) (Duchefa, Belgium) agar medium at 32 seeds/plate, under LD conditions (16 h light/8 h dark, 20°C, 100 µE m^−2^ h^−1^, 70% relative humidity) and collected at the 1.04 developmental growth stage [130]. Rosette leaves and young inflorescences were collected from plants at the 1.04 and 6.00 developmental growth stages, respectively [130], cultivated in LD conditions (16 h light/8 h dark), at 20°C, in growth chambers. Two independent total RNA extractions were performed with the RTN-70 RNA miniprep Sigma kit, according to the supplier's instructions. Hybridization, microarray analysis and statistical analyses, based on two independent biological replicates and two dye-swaps (i.e. four arrays), were performed as previously described [129], [131]. The microarray data were deposited both at the ArrayExpress Archive database (http://www.ebi.ac.uk/arrayexpress) (accession numbers E-CAGE-109 and E-MEXP-802) and at CATdb (http://urgv.evry.inra.fr/CATdb/; accession number Project: RA05-06_LIF), according to the Minimum Information About a Microarray Experiment (MIAME) standards. The Bio-Array Resource for Plant Functional Genomics (http://www.bar.utoronto.ca/) and its Classification SuperViewer Tool (Provart & Zhu, 2003) based on the functional classifications from GO (January 5, 2010, file ATH_GO_GOSLIM.20100105.txt), was used to calculate normed frequencies of the classes, bootstrap standard deviation and p-values. EasyGO (Gene Ontology enrichment analysis tool, http://bioinformatics.cau.edu.cn/easygo/) [132] was also used.

### Chromatin immunoprecipitation analysis

The genomic LHP1:MYC construct carrying a 2404 bp 5′ region (from +1 transcription site) and an 1130 bp 3′ region (from the Stop codon) was used to transformed the *lhp1-4* mutant. Homozygous lines with one T-DNA insertion were analyzed by western blot analysis using monoclonal mouse anti-c-Myc (clone 9E10, Sigma-Aldrich, Ref. M4439).

ChIP assays were performed on 7-day-old *in vitro* seedlings using anti-H3K27me3 (Upstate Biotechnology, Ref. 07-449), anti-c-Myc (clone 9E10) or anti-H3 (Abcam, Ref. ab1791) antibodies, modified from Gendrel *et al.* [133]. Briefly, after plant material fixation in 1% (v/v) formaldehyde, tissues were homogenized, nuclei isolated and lysed. Cross-linked chromatin was sonicated using a water bath Bioruptor UCD-200 (Diagenode, Liège, Belgium) (30 s on/30 s off pulses, at high intensity for 12 min) and pre-cleared for 1 h at 4°C with 50 µL of Dynabeads® Protein A (Invitrogen, Ref. 100-02D). The complexes were immunoprecipitated with antibodies, overnight at 4°C with gentle shaking, and incubated for 1 h at 4°C with 50 µL of Dynabeads® Protein A. Immunoprecipitated DNA was then recovered using the IPure kit (Diagenode, Liège, Belgium) and analyzed by quantitative real-time PCR. An aliquot of untreated sonicated chromatin was processed in parallel for use as the total input DNA control. Three biological replicates were used for all CHIP assays and qPCR were performed at least in duplicate and produced similar results.

### Light, SEM and CLSM microscopy analyses

For light microscopy, samples were fixed and embedded in Technovit 7100 resin (Kulzer, Wehrheim, Germany) as described [134]. Five µm thick sections were obtained on a Leica RM2055 microtome, stained 2 min with 0.05% (w/v) Toluidine Blue O in 50 mM citrate buffer pH 4 (TBO) [135], shortly rinsed, dried, mounted in Isomount 2000 Labonord (Ref. 05547535) and observed using a Leica DMRXA2 microscope. Fresh samples were analyzed using a Hirox SH-1500 Tabletop scanning electron microscope (SEM). GFP and YFP fluorescence was assessed with an inverted Leica TCS-SP2-AOBS spectral confocal laser scanning (CLSM) microscope (Leica Microsystems, Mannheim, Germany).

## Supporting Information

Table S1 Sequences of primers used in this study.
(DOC)
