# Supplementary material for: Control of Flowering and Cell Fate by LIF2, an RNA Binding Partner of the Polycomb Complex Component LHP1
Source: PLoS One. 2011 Jan 31;6(1):e16592. doi: 10.1371/journal.pone.0016592 (PMC3031606; doi:10.1371/journal.pone.0016592)
Supplement: Table S1 — Sequences of primers used in this study. (DOC) [file pone.0016592.s001.doc]

Table S1: Sequences of primers used in this study.
